# Temperature-Dependent Conformation Behavior of Isolated Poly(3-hexylthiopene) Chains

**DOI:** 10.3390/polym14030550

**Published:** 2022-01-28

**Authors:** Sanwardhini Pantawane, Stephan Gekle

**Affiliations:** Biofluid Simulation and Modeling, Theoretische Physik VI, Universität Bayreuth, 95440 Bayreuth, Germany; Sanwardhini.Pantawane@uni-bayreuth.de

**Keywords:** poly(3-hexylthiopene), stiff, flexible, semi-flexible polymers, Lennard–Jones model polymers, molecular dynamics simulation

## Abstract

We use atomistic as well as coarse-grained molecular dynamics simulations to study the conformation of a single poly(3-hexylthiopene) chain as a function of temperature. We find that mainly bundle and toroid structures appear with bundles becoming more abundant for decreasing temperatures. We compare an atomistic and a Martini-based coarse-grained model which we find in very good agreement. We further illustrate how the temperature dependence of P3HT can be connected to that of simple Lennard–Jones model polymers in a vacuum. Upon adding solvent (THF) we observe the occurrence of a prominent swelling of the molecular size at a temperature of about 220 K. This swelling is in close agreement with the interpretation of recent spectroscopic experiments which allows us to explain the experimental observations by an increased frequency of bundle structures.

## 1. Introduction

Poly(3hexylthiophene) (P3HT) is a conjugated polymer widely used in optoelectronic devices like thin film-field effect transistors and organic photovoltaics because of its high field-effect mobilities and significant mechanical strength [1]. These favorable properties are intimately related to its molecular arrangement [2,3,4,5]. The basis for understanding this molecular arrangement is a solid understanding of the behavior of a single P3HT chain. In this respect, Heffner et al. [6] carried out dynamic light scattering experiments on isolated chains concluding that P3HT behaves as a flexible polymer. More recently, neutron scattering (SANS) experiments by the authors of [7] and viscosity measurements by the authors of [8] determined the persistence length from which they suggested to classify P3HT as a semiflexible polymer instead. Besides these, refs. [9,10] conducted single molecule spectroscopy experiments finding different chain conformations depending on the solvent quality. Raithel et al. [11] investigated local planarization effects and torsional order on the scale of a single emitting site. On the numerical side, in addition to their experimental work, ref. [9] conducted coarse-grained molecular dynamics (MD) simulations finding a highly anisotropic, ordered structure for regioregular P3HT at temperatures below the collapse transition. Schwarz et al. [12] found similar structures and investigated their role as precursors for multi-chain aggregates while Tapping et al. [13] used coarse-grained molecular dynamics simulations in combination with exciton modeling to predict absorption and emission spectra.

A recent series of spectroscopic experiments on highly monodisperse, regioregular P3HT by Panzer et al. [14,15] specifically focused on the detailed temperature dependence of the molecular arrangement. As the temperature was increased from 170 K to 300 K, ref. [14] noted a shift in the absorption spectra from blue to red. These results led to the interpretation that the collapse transition from a random coil at high temperatures into a more regular conformation at low temperatures proceeds via an intermediate more extended structure termed a “planarized swollen coil”. This interpretation was rationalized by the classical work of Kolinski et al. [16] who conducted grid-based Monte-Carlo simulations on semi-flexible model polymers and predicted a swelling of the polymer before the final collapse at low temperatures within a narrow parameter range. Interestingly, similar effects were also observed during film formation in P3HT by the same authors.

Motivated by these experiments, we here revisit the temperature-dependent single-chain behavior of P3HT using both atomistic as well as coarse-grained MD simulations. Our specific aim is to investigate explicitly the “planarized swollen coil” structure whose existence has been derived from an interpretation of the spectroscopic signatures in [14]. For this, we start off by MD simulations on the temperature-dependent collapse transition of a simple Lennard–Jones model system where we reproduce the conformations obtained in earlier works using Monte-Carlo systems [16,17,18,19]. In order to become more realistic, we then compare these model systems to MD simulations of P3HT using an atomistic force field based on the work in [20] in a vacuum. In the next step, we use these highly detailed simulations to benchmark a coarse-grained model based on the Martini force field [21], finding good agreement with the fully atomistic model. Finally, the coarse-grained model is computationally efficient enough to allow us to study the P3HT behavior in explicit THF solvent. Here, we indeed observe a molecular swelling at temperatures around 220 K which can be closely connected to the “planarized swollen coil” observed in the experiments by Panzer et al.

## 2. Simulation Models and Details

### 2.1. Molecular Dynamics Simulations

We use the MD simulation package GROMACS [22] (version 2018.1) for all simulations. The LINCS algorithm is used for constraining the bonds and a cutoff radius of 1.0 nm is used for the non-bonded potential. To maintain a constant temperature, we employ a velocity rescaling thermostat [23]. Periodic boundary conditions are applied in all three directions. One of the key quantities which we analyze in our work is the mean-squared radius of the gyration of a polymer averaged over time which is defined as

(1) $$\langle S^{2}\rangle =\frac{1}{n}\langle \sum_{i=1}^{n}{\left(r_{i}-r_{CM}\right)}^{2}\rangle$$

where $r_{i}$ is the position of monomer *i*, $r_{CM}$ is the center of mass of the polymer, and 〈〉 denotes an ensemble average over the trajectory of the MD run. Secondly, we calculate the maximum distance between the two monomers of the polymer and denote it with $D_{max}$. The errors bars denote the standard deviation of the sampling data.

### 2.2. Lennard–Jones Model Polymer

In the Lennard–Jones (LJ) polymer each monomer is modeled as a single LJ bead of the form

(2) $$U_{LJ}=4\epsilon \left[{\left(\frac{\sigma }{r}\right)}^{12}-{\left(\frac{\sigma }{r}\right)}^{6}\right]=\frac{C_{12}}{r^{12}}-\frac{C_{6}}{r^{6}}$$

$C_{12}$ was fixed at $10^{-6}\frac{\mathrm{kJ}}{\mathrm{mol}\;{\mathrm{nm}}^{12}}$ while the $C_{6}$ values were varied in order to tune the polymer stiffness as described below. Neighboring monomers are connected to each other with a harmonic potential $U_{bond}$ of the form

(3) $$U_{bond}=\frac{1}{2}k^{b}{\left(r-b\right)}^{2}$$

The equilibrium distance *b* between two monomers was set to 0.1 nm. Angle potentials had the form

(4) $$U_{angle}=\frac{1}{2}k^{\theta }{\left(\theta -\theta_{0}\right)}^{2}$$

with the equilibrium angle $\theta_{0}=180^{\circ }$ and the bending stiffness used as a tuning parameter.

The stiffness of the polymer was tuned in two different ways. In the first method, we fix $k^{\theta }=95.0\frac{\mathrm{kJ}}{\mathrm{mol}\;{\mathrm{rad}}^{2}}$ and vary the attractive non bonded parameter $C_{6}$ with the stiffest polymers having the most repulsive potential $\left(C_{6}=0.0000\frac{\mathrm{kJ}}{\mathrm{mol}\;{\mathrm{nm}}^{6}}\right)$ and the most flexible polymers having the most attractive potential $\left(C_{6}=0.001\frac{\mathrm{kJ}}{\mathrm{mol}\;{\mathrm{nm}}^{6}}\right)$. In the second method, we fix $C_{6}=0.00045\frac{\mathrm{kJ}}{\mathrm{mol}\;{\mathrm{nm}}^{6}}$ and vary the bending potential $k^{\theta }$ from $5.0\frac{\mathrm{kJ}}{\mathrm{mol}\;{\mathrm{rad}}^{2}}$ to $250.0\frac{\mathrm{kJ}}{\mathrm{mol}\;{\mathrm{rad}}^{2}}$ to tune the polymer stiffness.

In all LJ model simulations, the time step was set to 1 fs, and the simulations for T≤ 600 K were carried out 50 times with different random initial conformations for 5 ns each. The random initial conformations were extracted from a simulation run at a high temperature (for LJ polymers we used 400 K), where the polymer essentially performs a random walk. For T>600 K we did single simulations of 200 ns. The first 500 ps were discarded for analysis. In the simulations with varying bending potential, $\langle S^{2}\rangle$ is averaged over single simulation runs of 200 ns each for every temperature.

### 2.3. Atomistic Poly(3-hexylthiopene) Model

Bhatta et al. [20] developed a force field adjusted to P3HT which we use in our work. Departing from the basic OPLS-AA force field [24], they carried out large-scale DFT calculations (B3LYP/6-31+G(d,p)) with the explicit treatment of all atoms to investigate the fully relaxed equilibrium structures of P3HT oligomers up to 10 monomer units. This approach allowed them to achieve the long-chain convergence limit for the torsional parameters. With their force field, Bhatta et al. were able to reproduce experimental findings in a bulk system such as packing structure, torsional angles of the backbone, and the hexyl side chains. The fact that this force field was designed for long chains makes it particularly suitable for our investigations. As discussed in recent in-depth force field comparisons by the authors of [25,26,27], many other force fields, in contrast, are designed rather for short oligomers. Here, we therefore adjusted the force field of Bhatta et al. to form long, but finite chains of P3HT by adding uncharged hydrogen atoms at the end.

**Figure 1:**
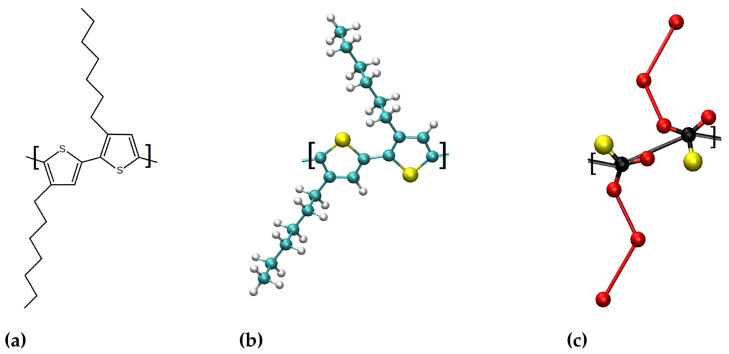
Schematic illustration of P3HT in (**a**), all-atom model in (**b**) and 6-site Martini CG in (**c**).

Schematics showing the central part of the planar P3HT are shown in Figure 1a. Figure 1b illustrates the structure of P3HT in the all-atom model. The validation of the force field was done by reproducing the system mentioned in [20] and studying the torsional angle populations, which is shown in Section S2 of the supporting information.

Similar to the LJ polymer in the previous section, the results below 600 K are averaged from 50 simulations of 5 ns each, and for above 600 K they are extracted from 125 ns NVT simulations with a timestep of 0.5 fs. Again, the first 500 ps of the simulations were discarded for analysis. Constraint algorithms, thermostat scaling, and periodic boundary conditions were kept the same as for the LJ polymers. Detailed results are provided in Section S2 of the supporting information.

### 2.4. Coarse Grained Martini Poly(3-hexylthiopene) Model

We adopted a coarse-grained (CG) model for P3HT directly from the work in [21]. The force field used follows the one created by Lee and Pao [28] and is based on the coarse-grained Martini force field [29]. The schematic representation of coarse-graining of the atomistic model with six beads per monomer is shown in Figure 1c. The distance between the two P3HT rings used in this model is 0.38 nm. The details of the model validation can be found in [21]. Plain cut-off was used with neighborlist radius and coulomb cut-off radius set to 1.0 nm. A velocity rescaling thermostat was used to keep the temperature constant. For temperatures below 600 K, 50 NVT simulation runs were done for 5 ns, and for above 600 K we did single simulation runs of 250 ns with 5 fs timestep and LINCS constraints. The initial 500 ps of the simulations were discarded when calculating the properties.

The CG Martini model of solvent tetrahydrofuran (THF) contains one bead per molecule and is taken from Patti et al. [30].

For the solvent simulations, we added 5000 THF molecules in a box of size (78.5 nm)${}^{3}$, pressure equilibration reduced to about (14 nm)${}^{3}$ at 300 K (further details are provided in supporting information Section S9). We used a Nosé–Hoover thermostat with $\tau_{t}=0.4$ ps and an isotropic Parrinello–Rahman barostat with $\tau_{p}=1.0$ ps and a reference pressure of 1.0 bar. The compressibility was set to 4.5 × 10${}^{-5}$ bar ${}^{\left(-1\right)}$ again, for all the temperatures below 600 K, we did 50 simulations each and the initial configurations of the polymer were extracted from the runs carried out at 2000 K (temperature well within the random walk limit). After the energy minimization step, we did 100 ps NVT simulation, followed by 2000 ps NPT equilibration runs. The system was then simulated for another 10 ns using NPT ensemble which was used for trajectory analysis. We found that 78 nm seems very large for 5000 THF.

## 3. Results

### Lennard–Jones Model Polymers

**Figure 2:**
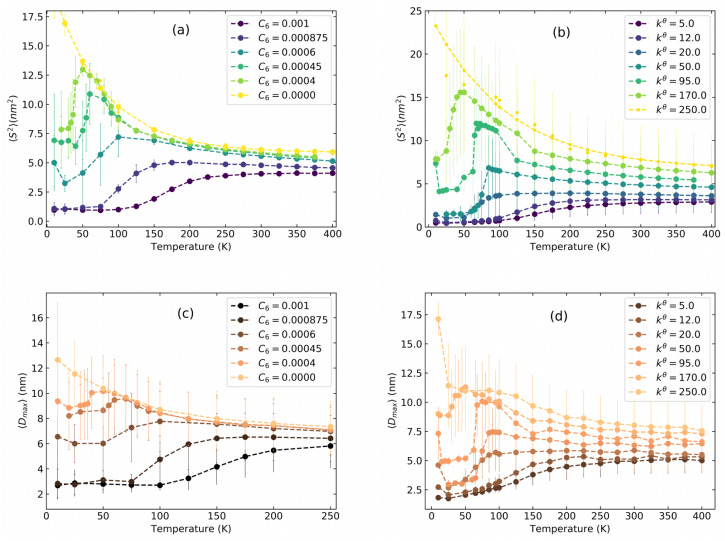
(**a**) Mean squared radius of gyration $\langle S^{2}\rangle$ as a function of temperature for a LJ polymer with n=200. The stiffness is tuned by the non bonded parameter $C_{6}$ in $\frac{\mathrm{kJ}}{\mathrm{mol}\;{\mathrm{nm}}^{6}}$. (**b**) Similar to (**a**), but the stiffness is tuned by varying the bending potential $k^{\theta }$ in $\frac{\mathrm{kJ}}{\mathrm{mol}\;{\mathrm{rad}}^{2}}$. (**c**) Average maximum distance between two monomers $D_{max}$ as a function of temperature for an LJ polymer where the stiffness is set by $C_{6}$. (**d**) Similar to (**c**) but tuning the bending potential.

In order to provide the necessary background for the P3HT investigations to be presented below, we start with a brief study of Lennard–Jones model polymers. Following [16], we plot in Figure 2a,b the radius of gyration $\langle S^{2}\rangle$ defined in Equation (1) for a polymer with N=200 monomers. The maximum distance between two monomers of a polymer averaged over time $D_{max}$ is presented in Figure 2c,d. As detailed in Section 2.2 above, the stiffness of the polymer can be tuned in two ways: in Figure 2a,c the attractive part of the LJ potential tunes the stiffness while in Figure 2b,d we tune the bending potential.

Going from right to left in Figure 2a, *stiff* polymers ($C_{6}=0$) show increasing $\langle S^{2}\rangle$ with decreasing temperature whereas *flexible* polymers ($C_{6}\geq 0.0007$) show decreasing $\langle S^{2}\rangle$ with decreasing temperature. In between these two regimes, some chains ($C_{6}\;=\;0.0004,0.00045,0.0006$) first exhibit increasing $\langle S^{2}\rangle$ with decreasing temperature followed by a sudden drop. These chains are termed *semi-flexible* polymers. The collapsed state minimizes the surface energy but at the cost of high bending energy. Indeed, a similar behavior is observed in Figure 2b, where the stiffness of the polymer is tuned by varying the bending potential. Taken together, Figure 2a,b clearly show that MD simulations confirm the intermediate swelling of the polymers before the low-temperature collapse predicted by the simpler grid-based Monte-Carlo models in [16]. The radius of gyration depending on the polymer length is compared to the classical theory of Flory [31] in Section S1 of the supporting information.

Much like in Figure 2a,b, *flexible* polymers show decreasing $D_{max}$ with decreasing temperature in Figure 2c,d. *Stiff* polymers have increasing $D_{max}$ with decreasing temperature whereas *semiflexible* polymers have increasing $D_{max}$ with decreasing temperature followed by a drop. Thus, the maximum extension of the polymer provides an additional tool to separate the various conformations. It will become an important basis for further analysis of P3HT polymers.

**Figure 3:**
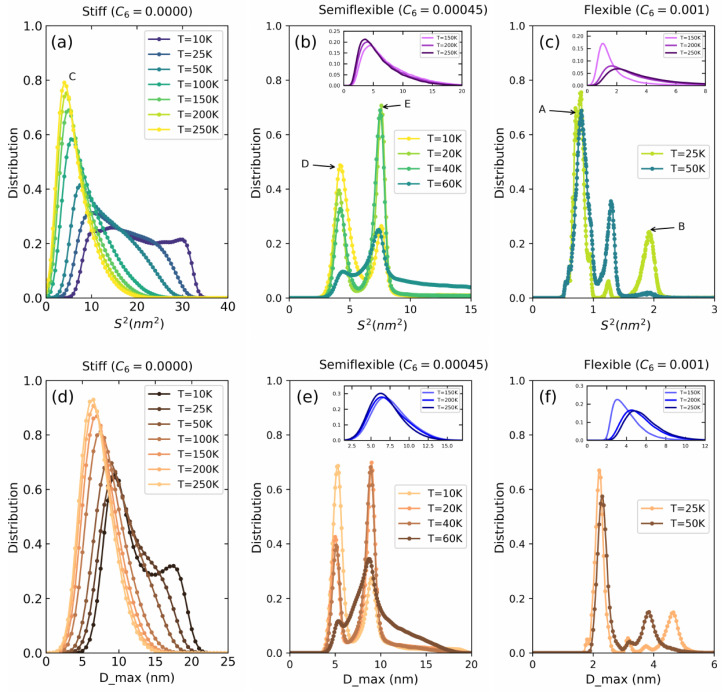
Distribution of $S^{2}$ for (**a**) stiff ($C_{6}=0.0000$), (**b**) semiflexible($C_{6}=0.00045$), and (**c**) flexible ($C_{6}=0.001$) polymers. Distribution of $D_{max}$ (**d**) stiff ($C_{6}=0.0000$), (**e**) semiflexible ($C_{6}=0.00045$), and (**f**) flexible ($C_{6}=0.001$) polymers.

Having presented the overall nature of $D_{max}$ and $\langle S^{2}\rangle$, we will now relate these insights to the temperature-dependent behavior for stiff, semi-flexible, and flexible polymers. As examples for these types, we select $C_{6}=0.0000$ (stiff), $C_{6}=0.00045$ (semi-flexible), and $C_{6}=0.001$ (flexible). Figure 3a represents the histograms for $\langle S^{2}\rangle$ and Figure 3b represents the histograms for $D_{max}$ at different temperatures.

**Figure 4:**
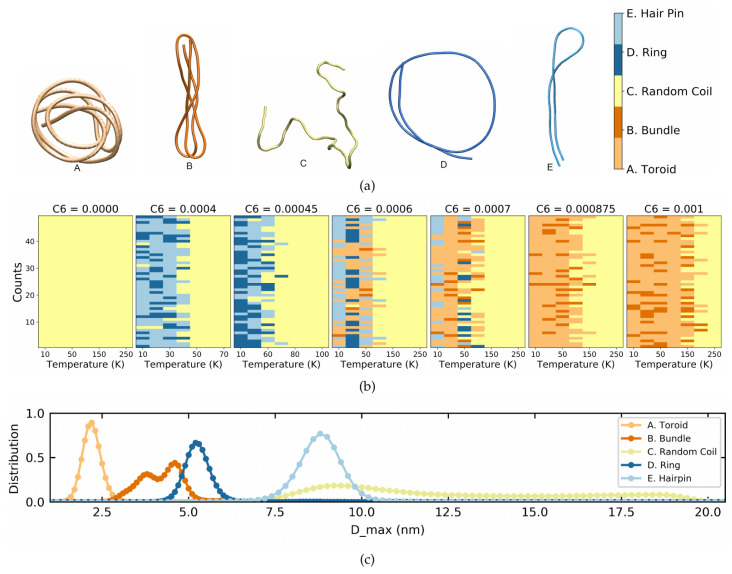
(**a**) From left to right illustration of toroid, bundle, random coil, ring and hairpin. (**b**) Color coded final conformations at a range of temperature for 50 simulations at different $C_{6}$ values. (**c**) $D_{max}$ distribution of the above five mentioned structures.

The names of the structures that will now be used for further discussion are shown in Figure 4a, which illustrates the different forms attained by the polymer. Going from left to right, the compact circularly wound form is labeled as a toroid (A), the multiple folded elongated structure is a bundle (B), the random coil is (C), a single circularly wound form is termed a ring (D), and finally a single folded elongated structure is termed a hairpin (E). The corresponding colors are light orange, dark orange, yellow, dark blue, and light blue, respectively. The nomenclature of these conformations is based on various previous studies [18,32,33,34] with whom we compare in Section S3 of the supporting information.

Starting with the distributions for the stiff polymer in Figure 3a, we see a peak at 5 ${\mathrm{nm}}^{2}$ at the highest temperature (250 K) with a tail towards higher radii. This structure corresponds to a random open coil that opens and closes rapidly and is thus marked with the label C. As we lower the temperature from 250 K to 100 K and further down to 75 K, we see a shift towards higher radii and a broadening of the distribution. At the lowest temperature of 10 K, a clear second peak at about 28 ${\mathrm{nm}}^{2}$ has appeared which corresponds to a stiffened and more elongated coil. This transition from rapidly opening and closing random coil to a more rigid coil gives rise to the an exponential increase in the $\langle S^{2}\rangle$ curve in Figure 2a,b for stiff polymers. The maximum extension exhibits a very similar behavior as shown in Figure 3d.

Semiflexible polymers are exceptionally interesting because of their swelling-before-collapse transition. When going from higher to lower temperatures, semiflexible polymers at first exhibit an increase in $\langle S^{2}\rangle$ due to a stiffening of the coil similar to the stiff polymers. However, at a certain temperature (50 K <T< 75 K) a collapse to either hairpins or rings is observed.

To study this collapse in more detail, Figure 3b shows the distribution first at temperatures below the collapse. At 10 K, this allows us to identify two clear peaks at 4.1 ${\mathrm{nm}}^{2}$ and 7.8 ${\mathrm{nm}}^{2}$ marked with the labels D and E, respectively. These confirm that the probability of forming rings and hairpins exists at all temperatures. As we increase the temperature from 10 K to 60 K, the peak at 4.1 ${\mathrm{nm}}^{2}$ decreases and the one at 7.8 ${\mathrm{nm}}^{2}$ increases indicating a shift in probability of the polymer for forming rings at low temperature (10 K) and hairpins at relatively higher temperatures (60 K). Above 70 K, the polymer forms an open random coil with a wide $\langle S^{2}\rangle$ distribution as shown in the inset of Figure 3b. Again, the $D_{max}$ distribution in Figure 3e exactly mirrors this behavior.

Flexible polymers shown in Figure 3c, in general, behave similarly to semiflexible polymers, however, their interplay at lower temperatures is between a toroid and a bundle rather than a ring and a hairpin. Therefore, the values of $\langle S^{2}\rangle$ are in general smaller. For temperatures above 150 K, we again observe a wide distribution corresponding to the random open coil conformation. At low temperatures (10–50 K) the peaks appear at 0.8 ${\mathrm{nm}}^{2}$ (marked with label A for toroids), 1.2 ${\mathrm{nm}}^{2}$, and 2.0 ${\mathrm{nm}}^{2}$ (marked with label B for bundles) which indicates the existence of both toroids and bundles as well as an unstable state between the two. Quantitalively similar behavior is reproduced by the histograms of $D_{max}$ in Figure 3f.

As suggested by the $\langle S^{2}\rangle$ distributions, the final conformation that a polymer attains depends on its initial conformation. In order to properly account for this behavior, multiple simulations with different initial conditions were carried out for each set of parameters. By visual inspection and comparison to the generic structures in Figure 4a, we then determined the type of conformation for each simulation. Figure 4b shows color coded final conformations for 50 simulations at each $C_{6}$ value and temperature. This allows visualizing the transition from flexible to semiflexible to stiff polymers by the gradual change from orange to blue to yellow region, respectively.

Furthermore, it allows us to study the $D_{max}$ distributions within each conformation. For this, we combine all conformations of a given type (A–E) independent of their $C_{6}$ and temperature and plot the corresponding distributions in Figure 4c. Clearly, the toroid is the most compact form with a $D_{max}$ peak at 2 nm, followed by the bundle at 3.75 nm, the ring at 5.3 nm, and the hairpin at 8.8 nm. The random coil is a very unstable structure with $D_{max}$ ranging from 7.5 to 20 nm.

## 4. Poly(3-hexylthiopene) (P3HT)

### 4.1. Atomistic P3HT

We now turn to study a realistic, atomistically resolved model for a single P3HT chain with 200 monomers. In this subsection, simulations are carried out in a vacuum mimicking a very bad solvent as detailed in Section 2.3.

**Figure 5:**
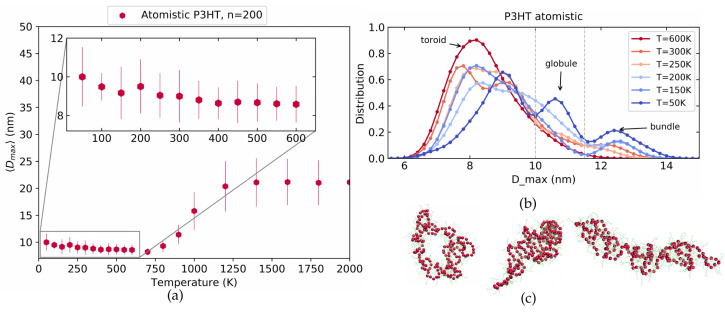
The maximum distance between monomers for the atomistic P3HT model in vacuum at different temperatures. (**a**) The averaged $D_{max}$ as a function of temperature shows a sigmoidal increase at very high and a slight increase towards low temperatures (inset). (**b**) The $D_{max}$ distributions at different temperatures exhibit three clear peaks corresponding to toroids, tight coils and bundles (from left to right) which are illustrated in (**c**).

Figure 5a shows the averaged $D_{max}$ at different temperatures. Most striking is the sigmoidal increase of $D_{max}$ with temperature which clearly reminds one of the LJ polymers in Figure 2. We note, however, that the sigmoidal increase happens at fairly high temperatures which are not usually accessible in experiments, as P3HT typically fully decomposes for temperatures above around 850 K [35]. In order to make the connection to experimental observations later, we, therefore, focus on the temperature range at and below 600 K which is enhanced in the inset. Interestingly, we here observe a slight but systematic swelling of the structure demonstrated by an increase in $D_{max}$ towards lower temperatures. To investigate this phenomenon further, we revert to the machinery established above for the LJ polymers and show in Figure 5b the $D_{max}$ distributions at different temperatures. At low temperatures, three clear peaks are observed. Visual inspection allows us to identify the left-most peak at about 8 nm with a toroid structure and the right-most peak at about 12.5 nm with a bundle. The corresponding conformations are shown in Figure 5c. In contrast to the LJ model polymer of Figure 3b, however, P3HT exhibits a prominent third peak at intermediate $D_{max}$ around 11 nm. This peak corresponds to a tight globular structure for which an example is also shown in Figure 5c. A more extensive set of conformations for these conformations is provided in Section S5 of the supporting information while similar data for $\langle S^{2}\rangle$ is shown in supporting information Section S4.1.

**Figure 6:**
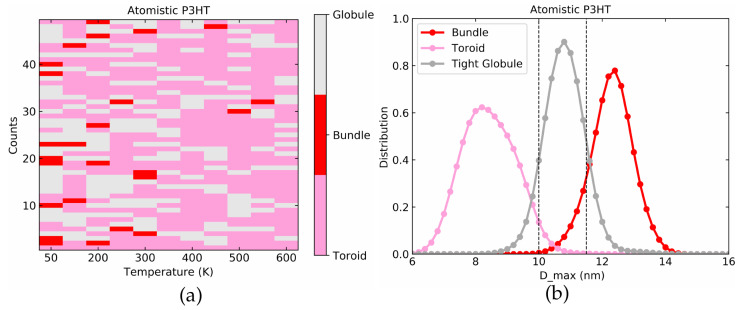
(**a**) Color coded final conformations at a range of temperature for 50 simulations for atomistic P3HT in vacuum. (**b**) $D_{max}$ distributions used for toroid, globules, and bundles.

Similar to the LJ polymers, we run a set of 50 simulations with different starting conditions for each temperature. Using $D_{max}$ from the final simulation frame and comparing to the dashed lines in Figure 5b allows us to classify the resulting structures into toroid, tight globule, and bundle. The result is shown in Figure 6a. Toroids are marked by pink and lie in the range of 0 nm $<D_{max}<$ 10.0 nm, globules are marked by grey and have 10.0 nm $<D_{max}<$ 11.5 nm while bundles have $D_{max}>$ 11.5 nm and are marked by red. As we decrease the temperature from 600 K to 50 K, the bundle conformations clearly become more frequent. However, for most of the temperatures below 300 K, both bundle and toroid structures coexist along with tight globule. Picking out all the simulations in the toroid, tight globule, and bundle regions individually we plot the $D_{max}$ distribution averaged over last 500 ps of each run in Figure 6b which underscores how each conformation directly corresponds to a distinct peak of $D_{max}$.

Finally, we note that we have not observed helical structures such as those shown in [25].

### 4.2. Martini P3HT in Vacuum

We now proceed with a similar analysis for our coarse-grained Martini model of P3HT, again starting in vacuum and using n=200. As detailed in Section 2.4, here each P3HT monomer is described by six beads.

**Figure 7:**
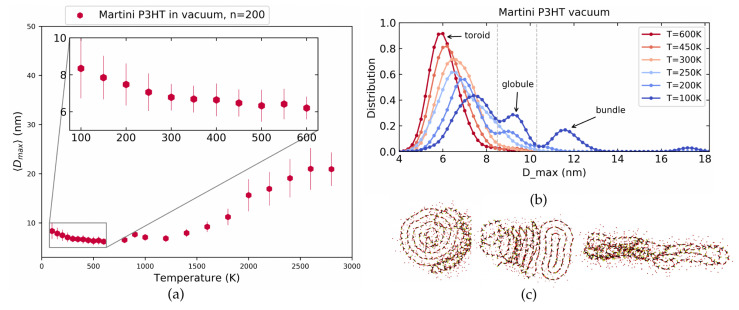
The maximum distance between monomers for the Martini model of P3HT in vacuum. (**a**) Averaged $D_{max}$ as a function of temperature. (**b**) Distributions at various temperatures. (**c**) Illustration of the corresponding conformations: toroids, tight globules and bundles.

Figure 7a shows averaged $D_{max}$ while Figure 7b displays the $D_{max}$ distributions at the different temperatures similar to Figure 5 above. In Figure 7a we again see a simple sigmoidal increase in $D_{max}$ for very high temperatures and a less pronounced, but still systematic swelling for decreasing temperatures within a realistic range of 600 K to 100 K. The $D_{max}$ distribution in Figure 7b is in good agreement with the atomistic model shown in Figure 5 above.

We notice three prominent peaks at low and a single strong peak at high temperatures. The transition between these peaks upon a change in temperature proceeds in a similar fashion as for the atomistic case with the only difference being that the Martini model overall exhibits slightly more compact structures than the atomistic model. Visual inspection of the corresponding real-space conformations allows identifying again the sequence of toroids, tight globules, and bundles as shown in Figure 7c. The borders between these phase as marked by the grey lines in Figure 7b are 0 nm $<D_{max}<$ 8.5 nm for toroids, 8.5 nm $<D_{max}<10.3$ nm for tight globules and finally $D_{max}>10.3$ nm for bundles. In fact, the geometric characteristics are even more pronounced than in the atomistic model. For additional conformations and $\langle S^{2}\rangle$ data, see supporting information, Sections S4.2 and S6.

**Figure 8:**
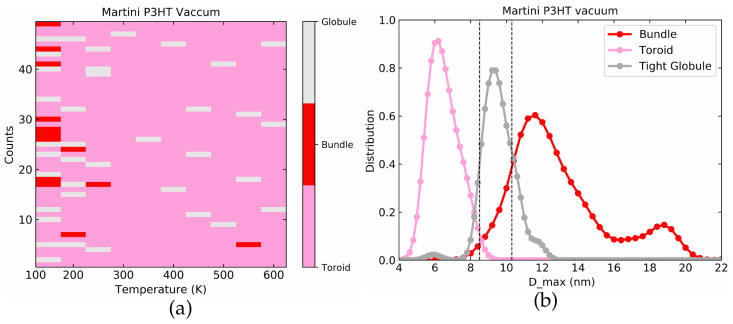
(**a**) Color coded final conformations at a range of temperature for 50 simulations for the Martini model of P3HT in a vacuum. (**b**) $D_{max}$ distributions used for toroid, globules, and bundles.

Figure 8a represents the color-coded outcome of the last frame of the 50 simulations run at each temperature with different initial conditions. Figure 8b shows the individual distribution of $D_{max}$ for toroids, globules, and bundles. Not surprisingly, both figures show very good agreement with the atomistic model in Figure 6.

### 4.3. Martini P3HT in THF Solvent

**Figure 9:**
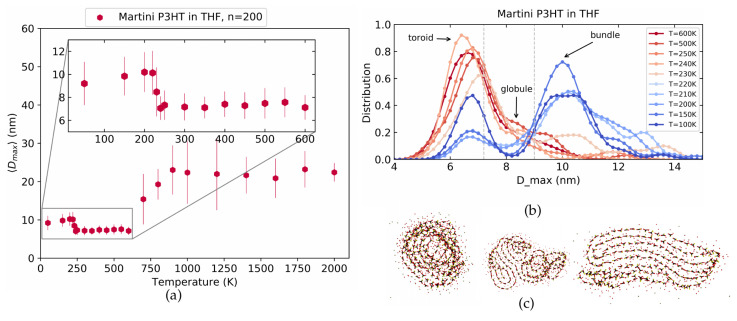
The maximum distance between monomers for the Martini model of P3HT in THF solvent. (**a**) Averaged $D_{max}$ as a function of temperature. (**b**) Distributions at various temperatures. (**c**) Illustration of the corresponding conformations: toroids, tight globules and bundles.

In order to match more closely a typical experimental situation such as the one in [14,15], we now study the behavior of P3HT chains in THF solvent. For the solvent, we use the Martini model described in the Methods Section 2.4 above. The maximum extension as a function of temperature shown in Figure 9a is similar to the previous cases in a vacuum with one notable exception: at about 220 K we observe a rather abrupt increase in $D_{max}$ (going from right to left) after which the values decrease again. This corresponds to a swelling of the molecule and is also reflected, albeit less strongly, in the radius of gyration shown in S4.3 of the supporting information.

**Figure 10:**
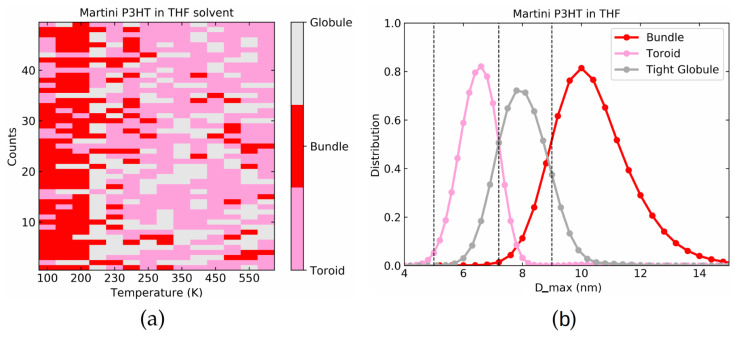
(**a**) Color coded final conformations at a range of temperature for 50 simulations for the Martini model of P3HT in THF solvent. (**b**) $D_{max}$ distributions used for toroid, globules and bundles.

Looking at the distributions in Figure 9b, we note as a further difference that the middle peak is no longer present. Nevertheless, the two prominent peaks at around 6 nm and 10 nm corresponding to toroids and bundles, respectively, are still clearly visible. The geometrical appearance of toroids and bundles is in general similar to the vacuum case as seen by the examples in Figure 9c as well as Section S7 of the supporting information. In Figure 10 we show the occurrence probabilities of toroid, bundles and globules for the 50 different initial conditions. Similar to the above two cases, the criteria for $D_{max}$ that was used for distinguishing these classes is shown by dashed gray lines in Figure 9b. Toroids lie in the range of $0<D_{max}\leq 7.2$ nm, globules within $7.2<D_{max}\leq 9$ nm, and bundles have dimensions $D_{max}>9$ nm. We note that one effect of the solvent is to increase the frequency of the bundle as compared to the vacuum case. As confirmed by the larger size of the bundle structure (see Figure 9b), this explains the sudden rise in $D_{max}$ at around 220 K.

As the situation in THF is the most experimentally relevant, we provide here two further characterizations of the observed structures: the π-π-stacking distance and the conjugation length. We compute the π-π-stacking distance within the bundle structure in which the chains fold back multiple times on themselves as clearly seen in Figure 9c. This backfolding is particularly important as it is connected to the electronic mobility in organic transistors by virtue of the π-π stacks. Following the analysis methods of [12], we found that the distance between the π-π stacked layers in our bundle conformations amounts to 4.73±0.23 nm. The slight deviation of this number from the experimental value may be attributed to the approximation of the thiophene ring as a spherical site in the CG model, which may overestimate the steric bulk of the ring in the π-π direction.

The conjugation length is an intrinsically electronic quantity, however, a first estimate can be obtained from the distribution of dihedral angles. For this, we assume that P3HT is broken into electronically isolated units (chromophores) if the inter-monomer dihedral angle exceeds a particular threshold value. Following previous estimates of conjugation lengths in simulations of P3HT [36,37] a threshold value of 40${}^{\circ }$ was chosen. The conjugation length is then given by the number of consecutive monomers whose angle lies below the threshold.

**Figure 11:**
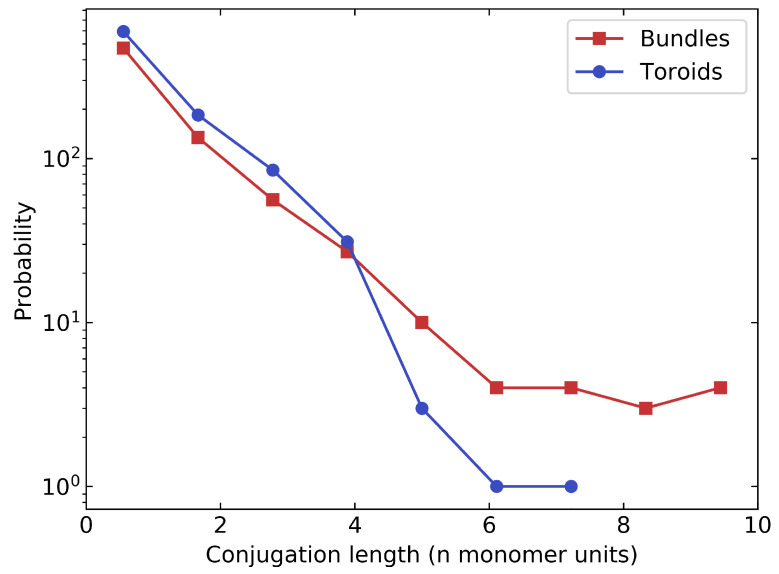
Distribution of conjugation lengths in toroids and bundles for P3HT in THF solvent.

The calculated distributions of conjugation lengths for bundles and toroids are shown in Figure 11. We find that large conjugation lengths in bundles are more than twice as likely as in toroids, which clearly demonstrates increased planarization of the polymer chains below 220 K.

## 5. Discussion and Conclusions

In summary, our detailed comparison shows that P3HT in vacuum inherits various typical characteristics of flexible polymers, namely the sigmoidal increase in $\langle S^{2}\rangle$ and $D_{max}$ upon increasing the temperature and the occurrence of toroids and bundles at low temperatures. Once immersed into THF solvent, however, new phenomena arise. Most prominently, we observe a rather sharp swelling of the molecule at a temperature of around 220 K.

This swelling behavior is indeed reflected closely in the recent series of spectroscopic experiments by Panzer et al. [14,15,38]. Panzer and coworkers worked on P3HT with molecular weights ranging from 5 kDa to 34 kDa corresponding to 30 to 200 monomers per polymer where they observed a change from red to blue in the absorption as well as emission spectra as they decreased the temperature. The spectroscopic signatures were interpreted that the polymer takes a random coil conformation at high temperatures and at lower energies the backbone and also the side chains planarize to form a crystalline planar structure which they assumed to be a “planarized swollen coil”. The change in the absorption spectra occurred via a bathochromic shift, suggesting an increased conjugation length which Panzer et al. connected to a swelling of the polymer before the final collapse into an ordered state (planarized) state. We hypothesize that this swelling is indeed connected to the rise of $D_{max}$ at 220 K in Figure 9a and to the increased conjugation length of bundle structures in Figure 11. Our simulations then furnish further insight into this structure which indeed is swollen (in terms of an increase in $D_{max}$) and in addition assumes a planar, bundle-like structure as shown in Figure 9c. The simulations thus allow us to interpret the swelling as an increased frequency of bundle structures that are more elongated than the compact globule and toroid structures observed at higher temperatures.

**Figure 12:**
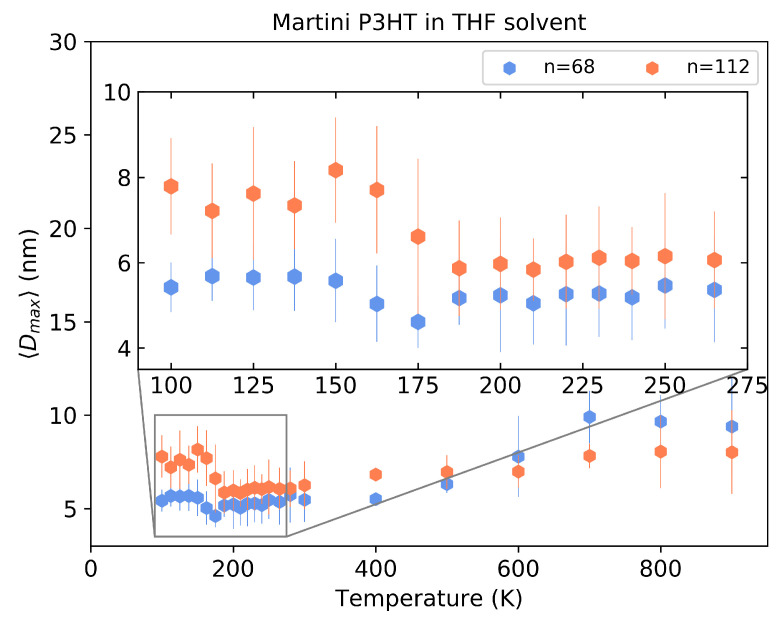
The maximum distance between monomers for the Martini model of P3HT with n=68 and n=112 in THF solvent.

To check the robustness of the observed effect, we conducted simulations with chain lengths of n=112 and n=68. $D_{max}$ as a function of temperature for these chains is shown in Figure 12. The transition temperature at which the swelling takes place is clearly visible around 185 K for n=112 while it is not so clearly distinguishable for the very short n=68 chain. This is in agreement with reports from the experiments in [15] that the critical temperature of P3HT chains in THF solvent decreases with decreasing chain length. Additional information about the distribution of $D_{max}$ is provided in Section S8 of the supporting information.

Interestingly, our simulations predict that this swelling is closely connected to the presence of the solvent THF which might make it a worthwhile effort to study the same transition in different in the future.

## Data Availability

Data is available upon request.

## Supplementary Materials

The following are available online at https://www.mdpi.com/article/10.3390/polym14030550/s1. Figure S1: Scaling of the mean-squared radius of gyration with the monomer number n shows ideal random walk behavior at high temperatures for the LJ model polymer. Figure S2: Snapshot of simulation box containing P3HT with 30 monomers and 32 repeats. (**a**) Side view with P3HT side chains going in and out of the plane. (**b**) Top view along the length of P3HT. (**c**) π−π stacking along with P3HT stacking along y-axis (**d**) Schematic of P3HT central portion. Figure S3: Torsional population at 300K for P3HT chains with n = 13, 20, 30 as a function of (**a**) α, (**b**) $\beta_{1}$ and (**c**) $\beta_{2}$ which show highest populations approximately at 180, 90 and 180 degrees respectively. Figure S4: Structural phase diagrams with respect to bending constant of our LJ model system. The conformation labels here follow the notation of Zierenberg et al (2016), and thus deviate from the ones used in the main text: R(random coil), H (hairpin), D3 (rod like bundle), K (toroids), C (globular). Figure S5: The state diagram of semiflexible polymers for chain stiffness (bending potential) as a function of inverse of temperature obtained from our MD simulations of an LJ model polymer. Figure S6: For Atomistic P3HT with n = 200: (**a**) $\langle S^{2}\rangle$ as a function of temperature. (**b**) $S^{2}$ distribution as a function of temperature. This figure corresponds to the data of Figure 5 of the paper. Figure S7: For Martini CG P3HT in vacuum with n = 200: (**a**) $\langle S^{2}\rangle$ as a function of temperature. (**b**) $S^{2}$ distribution as a function of temperature. This figure corresponds to the data of Figure 7 of the paper. Figure S8: For Martini CG P3HT in THF solvent with n = 200: (**a**) $\langle S^{2}\rangle$ i as a function of temperature. (**b**) $S^{2}$ distribution as a function of temperature. This figure corresponds to the data of Figure 9 of the paper. Figure S9: Typical toroids, globules and bundles found in the atomistic P3HT simulations at 300K. Figure S10: Typical toroids, globules and bundles found in the martini CG P3HT vacuum simulations at 200K-250K. Figure S11: Typical toroids, globules and bundles found in the martini CG P3HT in THF solvent at 300K. Figure S12: $D_{m}ax$ Distribution for different temperatures of Martini CG P3HT in THF solvent with (**a**) n = 68 and (**b**) n = 112. Figure S13: Box length for different simulations at 300 K.

## Abbreviations

The following abbreviations are used in this manuscript:

P3HT	Poly(3-hexylthiopene)
THF	Tetrahydrofuran
MD	Molecular Dynamics
ATB	Automated Topology Builder and Repository
