# Supplementary material for: Temperature-Dependent Conformation Behavior of Isolated Poly(3-hexylthiopene) Chains
Source: Polymers (Basel). 2022 Jan 28;14(3):550. doi: 10.3390/polym14030550 (PMC8840214; doi:10.3390/polym14030550)
Supplement: Supplementary file 1 [file polymers-14-00550-s001.zip › polymers-1516814-supplementary.pdf]

Temperature-dependent conformation behavior of isolated Poly(3-hexylthiopene) chains : Supporting Information

Sanwardhini Pantawane and Stephan Gekle

S 1 Validation of Flory's Relation for random walk

The temperature at which a polymer reaches the behavior of an ideal random walk is given by the relation $\langle S^2 \rangle \propto n^{(6/5)}$.

To check this for our Lennard Jones polymers, we simulated polymers with $n = 50, 100, 200, 400$ and 600 monomers at various temperatures. In the figure below, we find that the relation is approximately reached at $T = 250$K and above for flexible polymers ($C_6 = 0.001$).

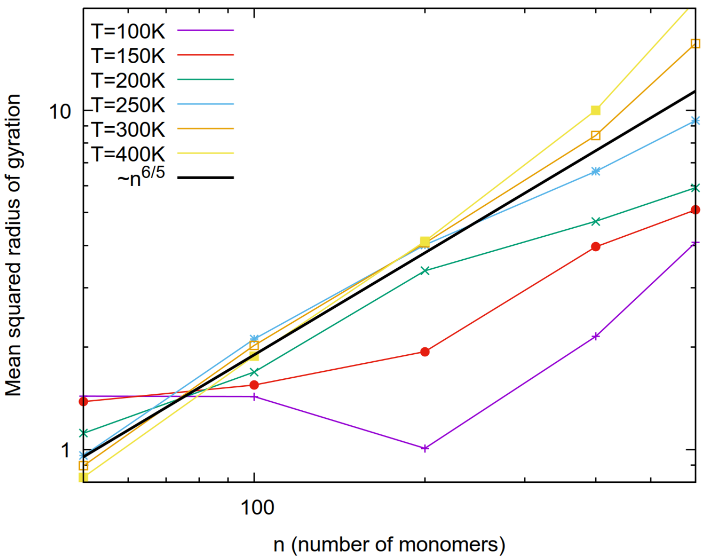

Figure S 1: Scaling of the mean-squared radius of gyration with the monomer number n shows ideal random walk behavior at high temperatures for the LJ model polymer.

S 2 Details and validation of the atomistic P3HT force-field

Our atomistic MD simulations are based on the force field presented by Bhatta *et al.* [1]. To validate our adpated forcefield, we reproduce Bhatta et al's system where the polymers were arranged in such a way that the length of the polymer extended along the x-axis, the side chains along the z-axis and stacking along the y-axis with 3.88 Å as the stacking distance, as shown in figure S 2. The simulations were performed starting

from 20K and were heated up to 550K with 20K increment up to 300K and 10K increment thereafter. The torsional angles were then comapred to their results.

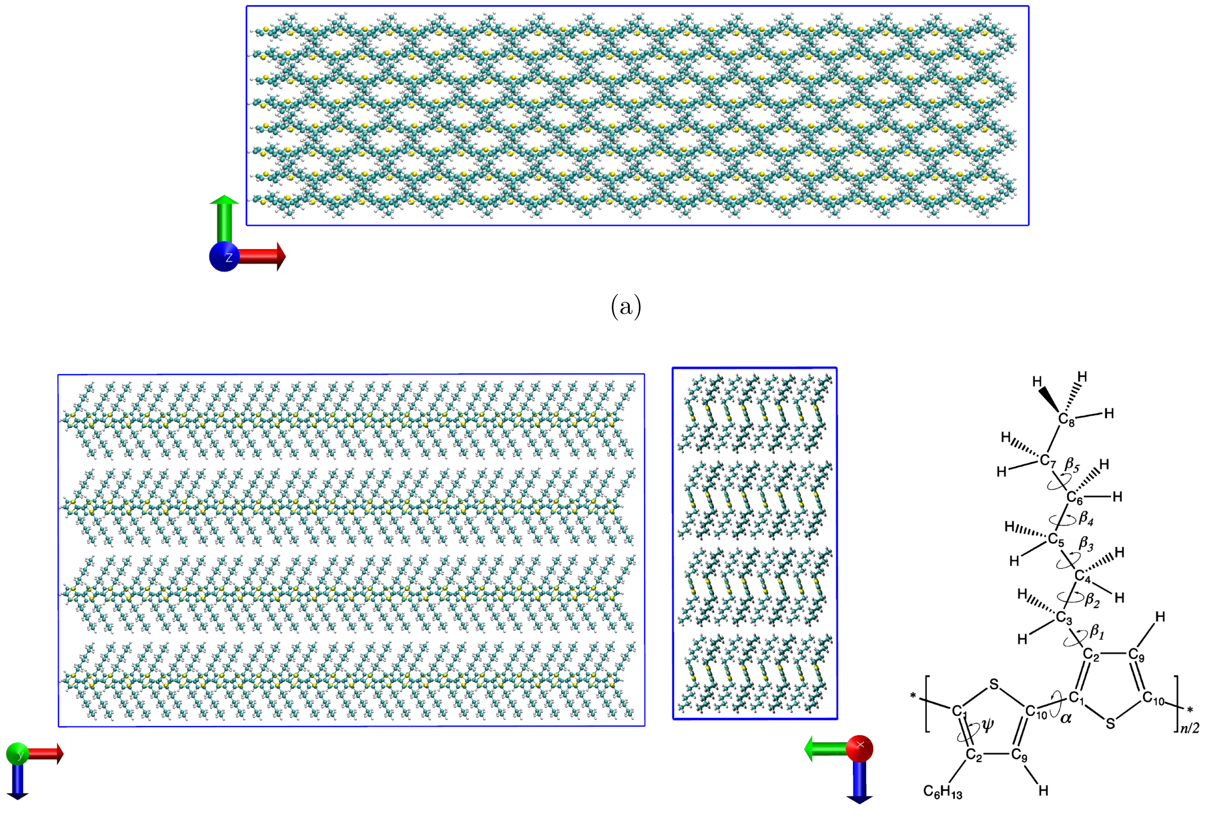

(a) (b) (c) (d)

Figure S 2: Snapshot of simulation box containing P3HT with 30 monomers and 32 repeats. (a) Side view with P3HT side chains going in and out of the plane. (b) Top view along the length of P3HT. (c) $\pi - \pi$ stacking along with P3HT stacking along y-axis (d) Schematic of P3HT central portion from [1]

We further validated the torsional angles α, β_1 and β_2 for an individual molecule as illustrated in figure S 2 (d). The torsional populations are shown in figure S 3. The maximum is at 180^0 and for $\beta_1$and β_2 at 90^0 and 180^0, respectively. This result matches with the results of [1] shown in figure S 3 (d).

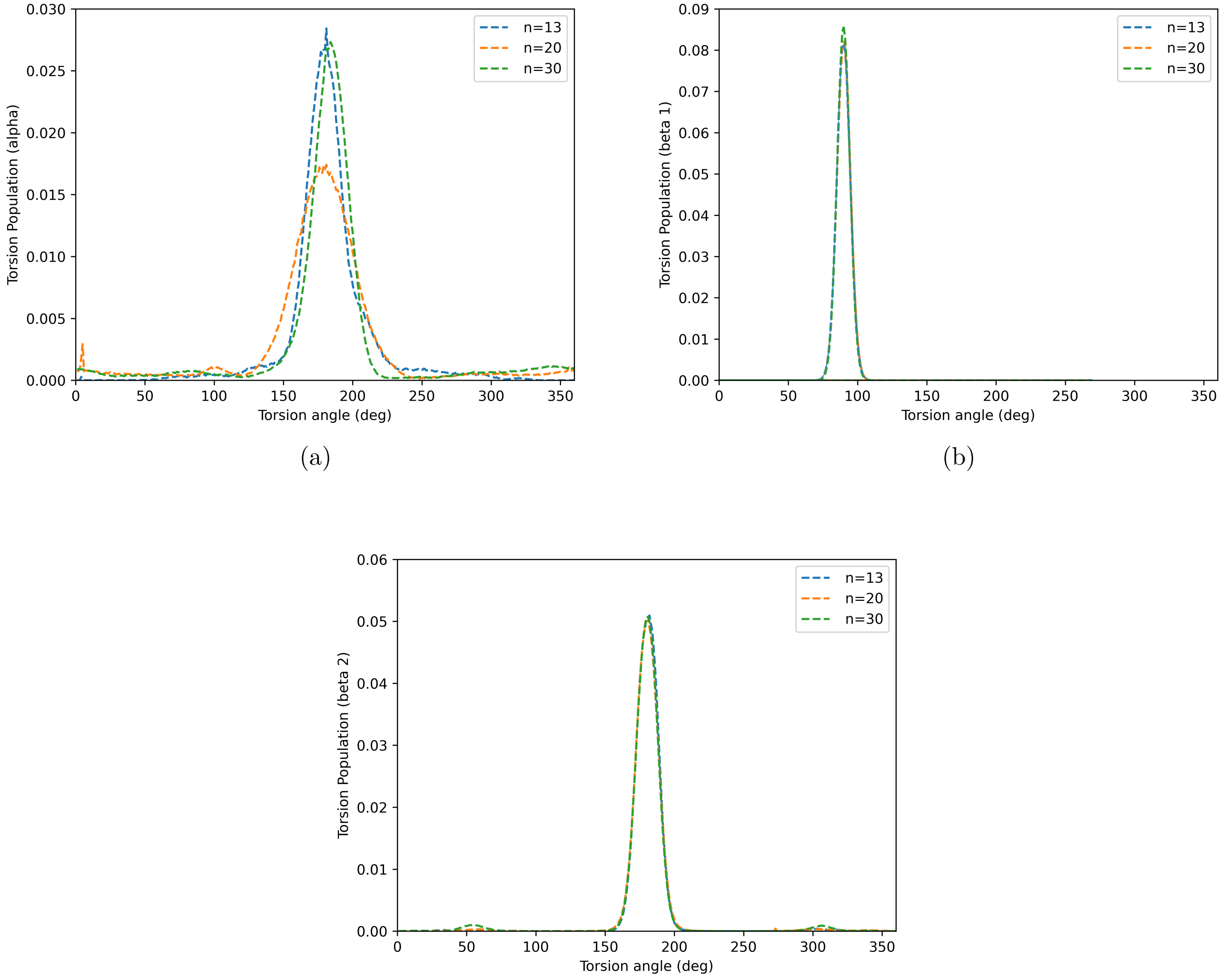

(a) (b) (c)

Figure S 3: Torsional population at 300K for P3HT chains with n=13, 20, 30 as a function of (a) α, (b)β_1 and (c) β_2 which show highest populations approximately at 180, 90 and 180 degrees respectively.

S 3 Validation of LJ Polymers

Figure S 4 represents a structural phase diagram for our LJ model as a function of the bending constant k^θ in equation (4) of the main text. The landscape clearly shows the division into the major regions: toroids, bundles, hairpins, random coils. The basic shape of the phase diagram agrees well with the ones observed by [4, 3] implying the robustness of our MD approach.

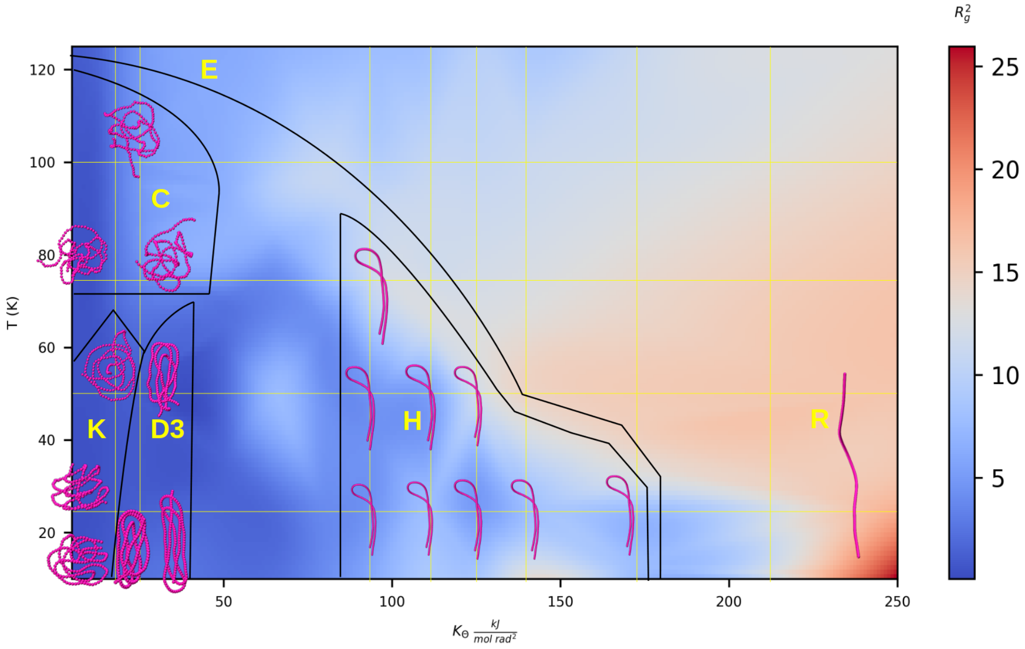

Figure S 4: Structural phase diagrams with respect to bending constant of our LJ model system. The conformation labels here follow the notation of [4] and thus deviate from the ones used in the main text: R (random coil), H (hairpin), D3 (rod like bundle), K (toroids), C (globular)

In a similar direction, figure S 5 shows the state diagram with inverse of temperature as a function of bending constant k^θ for semiflexible polymers, which also agress well with the results of [2].

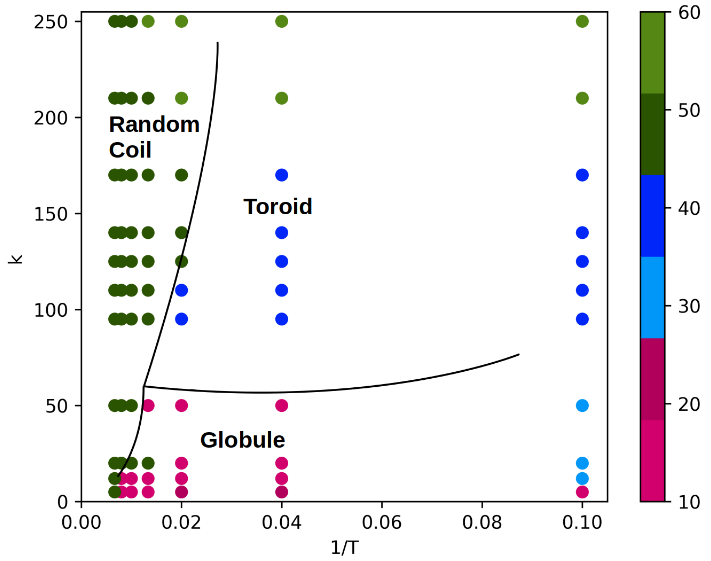

Figure S 5: The state diagram of semiflexible polymers for chain stiffness (bending potential) as a function of inverse of temperature obtained from our MD simulations of an LF model polymer agrees well with the one in [2]

S 4 Mean squared radius of gyration

S 4.1 Atomistic P3HT model

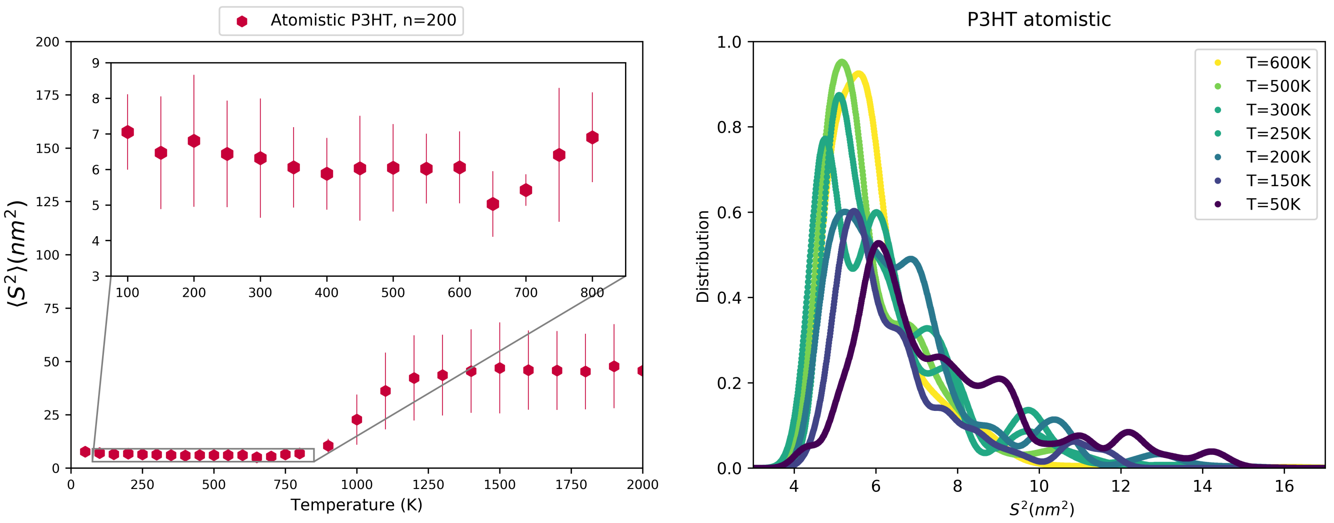

Figure S 6: For Atomistic P3HT with n = 200: (a) $\langle S^2 \rangle$ as a function of temperature. (b) S^2 distribution as a function of temperature. This figure corresponds to the data of figure 5 of the paper.

S 4.2 Martini CG P3HT model in vacuum

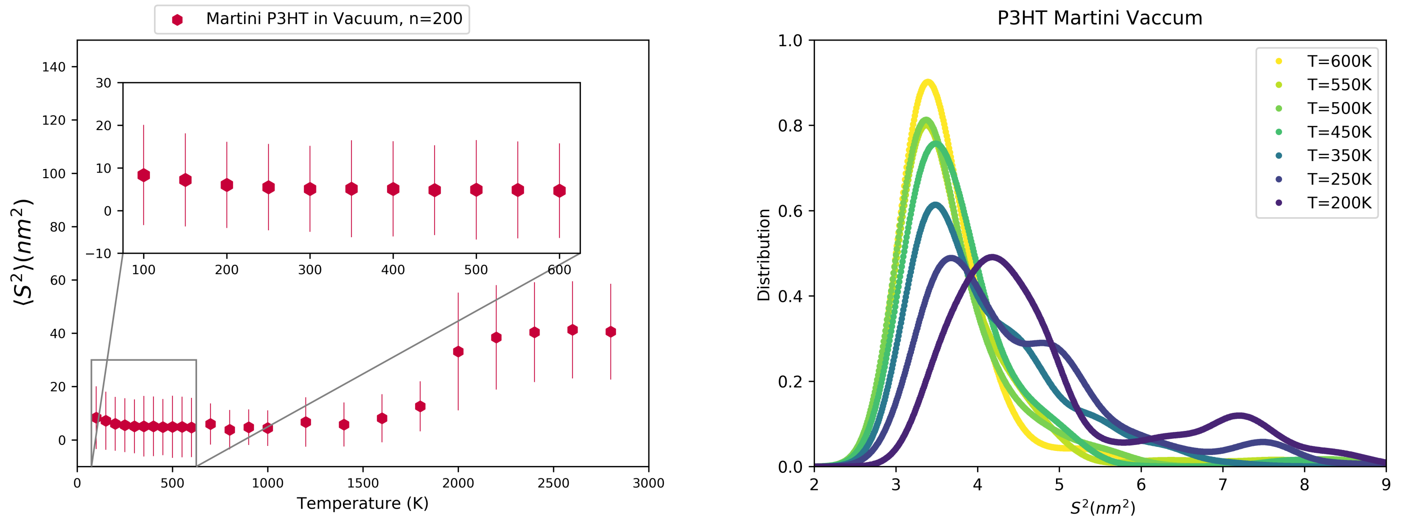

Figure S 7: For Martini CG P3HT in vacuum with n = 200: (a) $\langle S^2 \rangle$ as a function of temperature. (b) S^2 distribution as a function of temperature. This figure corresponds to the data of figure 7 of the paper.

S 4.3 Martini P3HT model in THF solvent

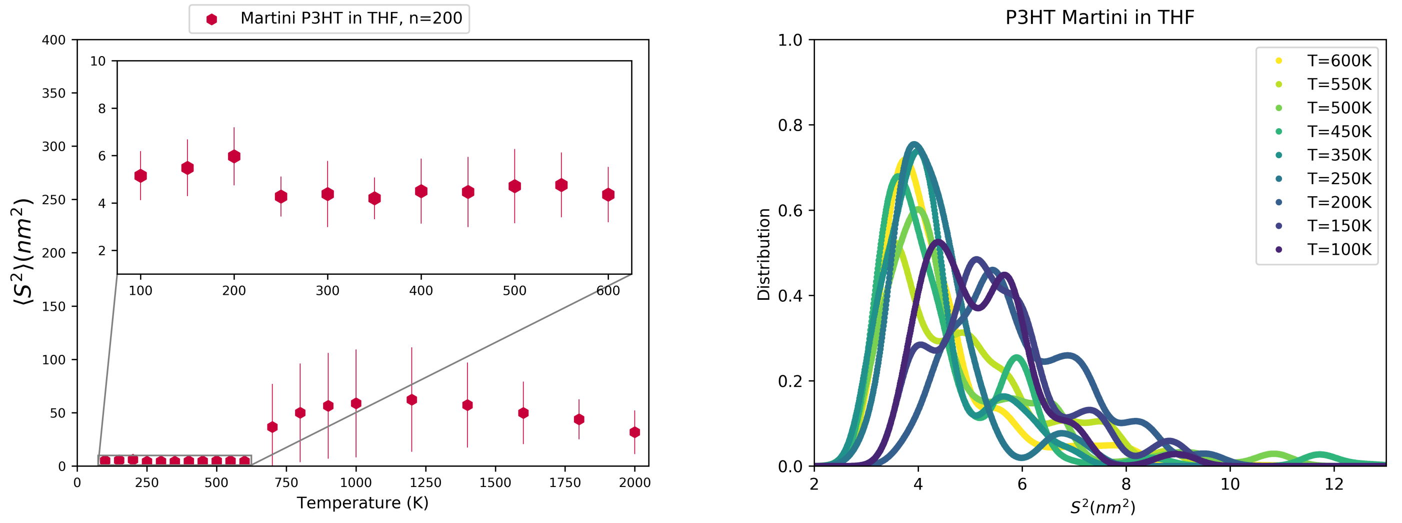

Figure S 8: For Martini CG P3HT in THF solvent with n = 200: (a) $\langle S^2 \rangle$ as a function of temperature. (b) S^2 distribution as a function of temperature. This figure corresponds to the data of figure 9 of the paper.

S 5 Atomistic Conformations

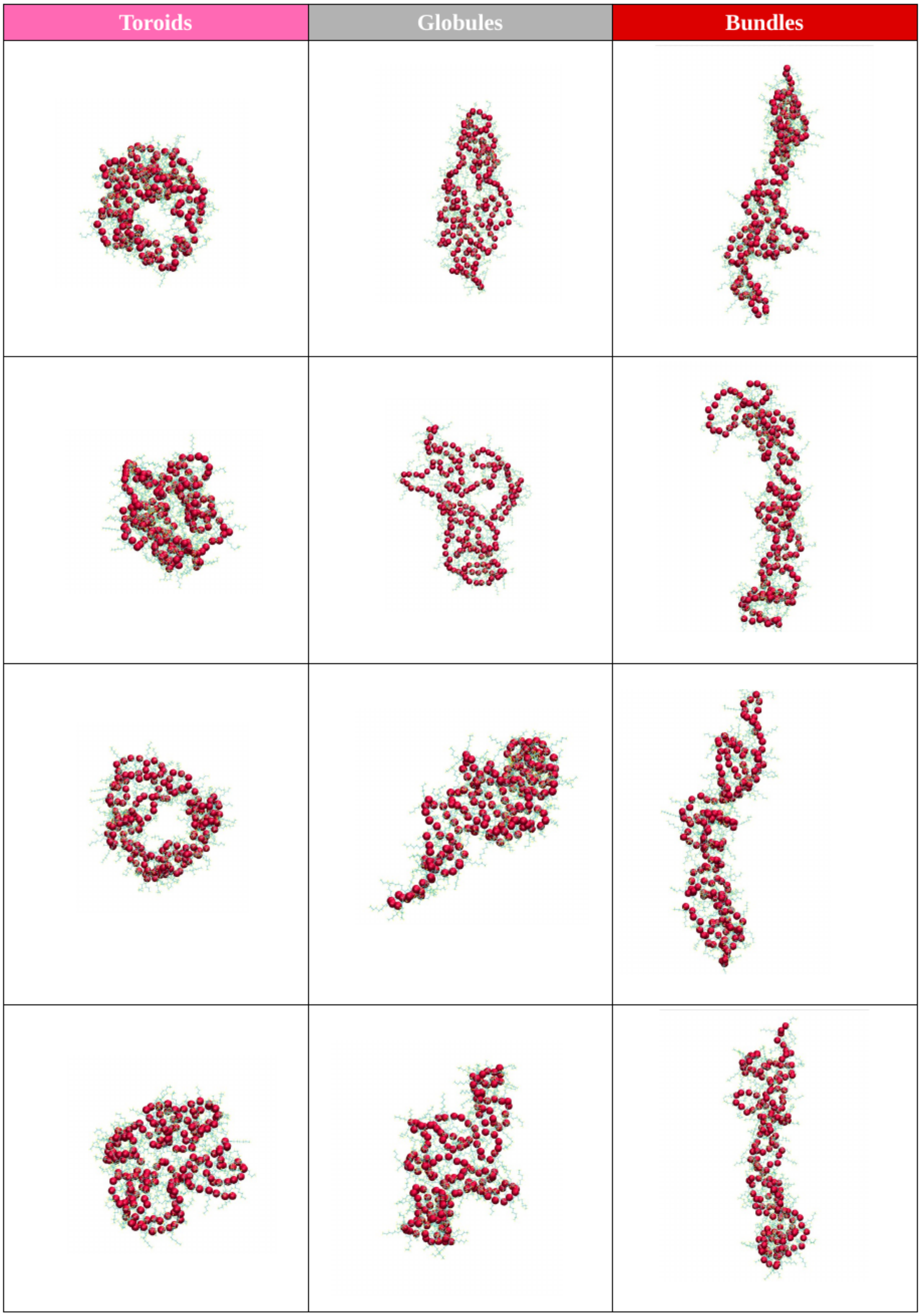

Figure S 9: Typical toroids, globules and bundles found in the atomistic P3HT simulations at 300K.

S 6 Martini CG Conformations in vacuum

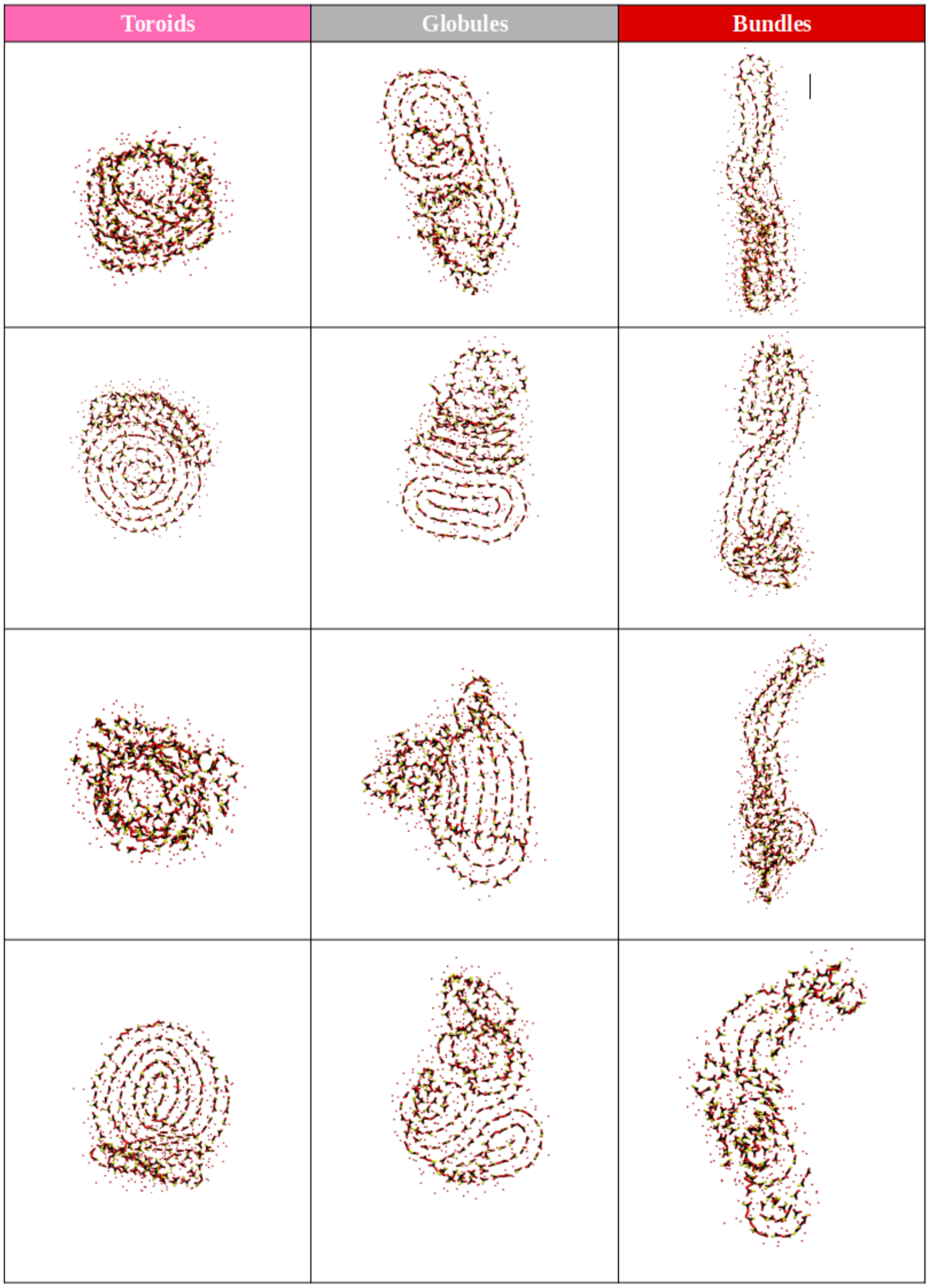

Figure S 10: Typical toroids, globules and bundles found in the martini CG P3HT vacuum simulations at 200K-250K.

S 7 Martini CG Conformations in THF solvent

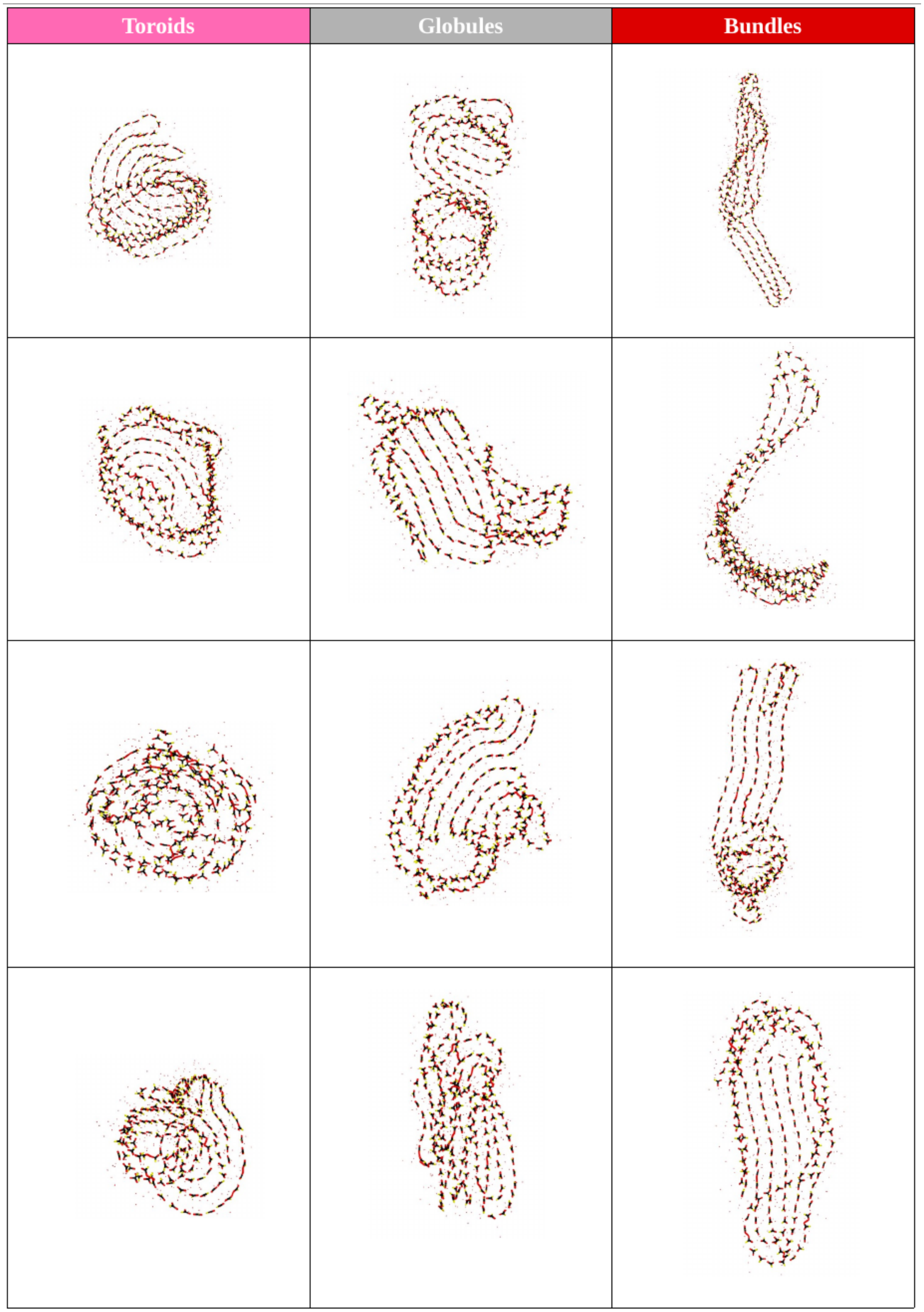

Figure S 11: Typical toroids, globules and bundles found in the martini CG P3HT in THF solvent at 300K.

S 8 Martini P3HT model in THF solvent with smaller chains

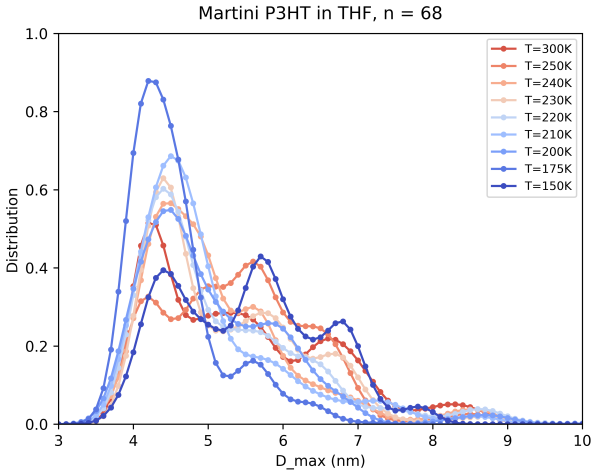

(a)

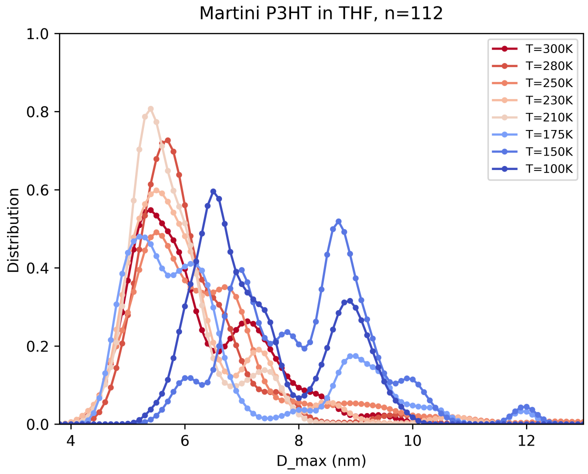

(b)

Figure S 12: D_{max} Distribution for different temperatures of Martini CG P3HT in THF solvent with (a) $n = 68$ and (b) $n = 112$

S 9 Martini P3HT model in THF solvent box size remark

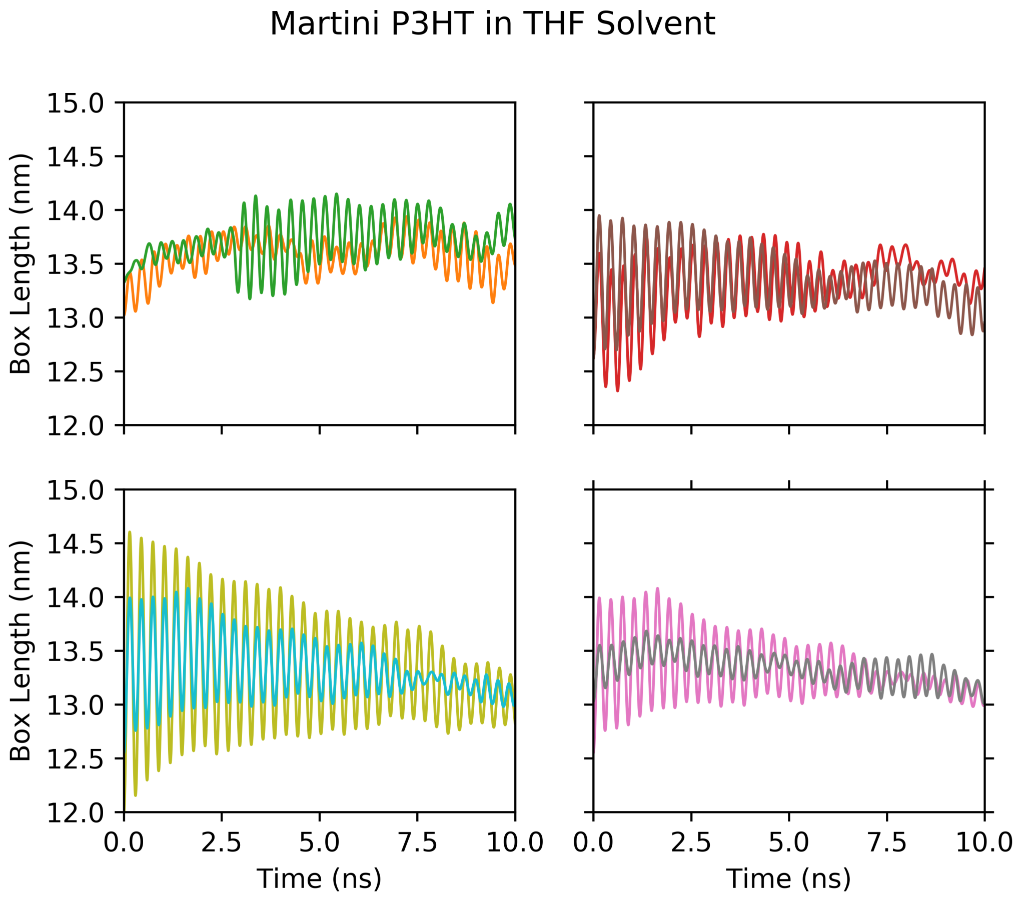

Figure S 13: Box length for different simulations at 300K

For a box with 5000 THF molecules with $\rho = 0.889 g/mL$ and 200 P3HT molecules, the cubic box dimensions should be $\approx 12nm$ however, in our case we have a bit bigger dimensions (average leading to $\approx 14nm$). This could be attributed to the side chains of P3HT and the coarse grained model.

References

[1] Ram S Bhatta et al. “Improved Force Field for Molecular Modeling of Poly(3-hexylthiophene)”. English. In: The Journal of Physical Chemistry B 117.34 (Aug. 2013), pp. 10035–10045.

[2] VA Ivanov et al. “Structures of stiff macromolecules of finite chain length near the coil-globule transition: A Monte Carlo simulation”. In: Macromolecular theory and simulations 9.8 (2000), pp. 488–499.

[3] Martin Marenz and Wolfhard Janke. “Knots as a Topological Order Parameter for Semiflexible Polymers”. English. In: Physical Review Letters 116.12 (Mar. 2016), pp. 128301–6.

[4] Johannes Zierenberg, Martin Marenz, and Wolfhard Janke. “Dilute Semiflexible Polymers with Attraction: Collapse, Folding and Aggregation”. English. In: Polymers 8.9 (2016), pp. 333–19.
